# Gender disparities in the development of clinical practice guidelines in Peru

**DOI:** 10.17843/rpmesp.2026.432.15175

**Published:** 2026-06-25

**Authors:** Fabian A. Chavez-Ecos, Patricia J. Vera-Maccha, Sandra S. Chavez-Malpartida, Leonardo J. Uribe-Cavero, Alexander M. Parra-Huaroto, Ana M. Avalos-Bautista, Miguel A. Chavez-Gutarra, Carlos Alva-Díaz, Wendy Nieto-Gutierrez

**Affiliations:** 1 Grupo de investigación NEMECS: Neurociencias, Metabolismo, Efectividad Clínica y Sanitaria, Universidad Científica del Sur, Lima, Peru.; 2 Red de Cardiología y Salud Pública (RCSP), Ica, Peru.; 3 Sociedad Científica de Estudiantes de Medicina de Ica (SOCEMI), Facultad de Medicina Humana, Universidad Nacional San Luis Gonzaga, Ica, Peru.; 4 Facultad de Medicina Humana, Universidad Nacional Mayor de San Marcos, Lima, Peru.; 5 Servicio de Neurología, Departamento de Medicina y Oficina de Apoyo a la Docencia e Investigación (OADI), Hospital Daniel Alcides Carrión, Callao, Peru.; 6 Unidad de Investigación para la Generación de Síntesis de Evidencia en Salud, Vicerrectorado de Investigación, Universidad San Ignacio de Loyola, Lima, Peru.

**Keywords:** Gender Equity, Practice Guideline, Women (source: MeSH NLM).

## Abstract

**Objective.:**

To explore gender disparity in the development of clinical practice guidelines (CPGs) in Peru.

**Materials and methods.:**

A descriptive and observational study was conducted. An electronic search for Peruvian CPGs published from 2015 until July 30, 2024, was performed. Microsoft Excel was used for data collection and RStudio for statistical analysis.

**Results.:**

177 CPGs published between 2015 and 2024 were analyzed that reported information related to the CPG development group (CDG). In general, the CDGs included 1527 men (58.8%) and 1070 women (41.2%) among clinical experts and methodologists. Men predominated as clinical experts, especially in specialties such as internal medicine and surgery; while women predominated as methodologists. The specialty with the highest male predominance was internal medicine (61.8%).

**Conclusions.:**

The study revealed lower female participation in the development of CPGs in Peru, as well as greater gender disparity in participation in the surgical field and in public institutions. The low participation of women in leadership positions influences this gap. Strategies are required to improve gender equity.

## INTRODUCTION

Globally, gender disparity in science remains latent ^1^. UNESCO (United Nations Educational, Scientific and Cultural Organization) identified in 2021 that the female gender represents 31.5% of researchers related to science, technology, engineering, and mathematics (STEM) worldwide, and 44.4% in Latin America and the Caribbean ^1^. This imbalance is driven by sociocultural factors, such as gender stereotypes, traditional roles, and a limited perception of belonging in STEM fields; phenomena that begin at an early age and can affect the self-sufficiency and confidence of women participating in these areas ^2^. Available data suggest an accentuated gender gap in developing countries, such as Peru. The National Census of Research and Development of Research Centers elaborated in 2016 showed that the female sex represented one-third of registered researchers ^3^. On the other hand, in the ranking of the 200 Peruvian researchers with the highest bibliometric indicators, it was identified that 15% were women, and only one was among the top ten positions ^4^.

This problem could impact key contexts such as processes linked to health decision-making; since they require the participation of a representative and inclusive group ^5^. The impact of gender disparity in processes such as the development of clinical practice guidelines (CPG) has been described in high-income countries. For example, Australia reported an underrepresentation of women in CPG development panels; 41% of them participated as members and 42% as lead developers ^6^.

In Peru, the methodological guidelines for CPG development were published in 2015, and their implementation has progressively consolidated in subsequent years. ^7^. Gender contribution data in these processes suggest a trend toward disparity. A 2023 study revealed that approximately 28% of participating members in CPG development were women; although this publication focused on cardiovascular disease guidelines ^8^.

Gender disparity has significant implications for the minority group, such as reduced opportunities for education, employment, and remuneration ^9^. Understanding the magnitude of this problem in health decision-making processes is important to identify and address the needs of the minority group from a gender-sensitive approach ^10^. This perspective is particularly relevant in a context like the Peruvian one, where gender violence and low female participation in leadership roles remain latent problems ^11^. The objective of this study is to analyze gender disparity in the participation of CPG development in Peru.

**KEY MESSAGES**

Motivation for the study. Gender disparity constitutes a little-explored topic in research and health decision-making processes. Promoting equality in the development of clinical practice guidelines (CPG) can guide the generation of recommendations that reflect an inclusive and diverse perspective.

Main findings. Gender disparity was identified in the Peruvian guideline development groups in relation to lower participation of women in roles as clinical experts and reviewers.

Public health implications. Raising awareness about gender disparity in the development of CPGs can lead to the adoption of an institutional approach with both public and private scope.

## MATERIALS AND METHODS

### Study design and population

A descriptive and observational study of Peru's CPG was developed based on the STROBE guideline (STrengthening the Reporting of OBservational studies in Epidemiology) ^12^ for observational studies to improve the reporting and quality of the study.

### Procedure, inclusion, and exclusion criteria

The selection of the CPG followed a structured process. First, all CPG published from 2015 onwards were considered (as this year was when the CPG began to use the GRADE (Grading of Recommendations Assessment, Development and Evaluation) methodology ^(^^7^). Second, a systematic electronic search was conducted in Google Scholar and platforms related to institutions and repositories, both in Spanish and English (supplementary table 1). Third, CPG were included from all medical topics related to internal medicine, obstetrics and gynecology, surgery, and pediatrics.

As an exclusion criterion, those CPGs were discarded that did not provide the nominal list of the members of the guideline development group (GDG) or whose information was insufficient to determine the gender and role of the participants. Additionally, if a CPG had multiple versions, the most recent version according to the publication date was selected. Information on the GDG was extracted into a Microsoft Excel 2019 spreadsheet. This process was carried out by four researchers (PJVM, LJUC, AMPH, and AMAB), and in case of discrepancies, a fifth author (CAD or WNG) resolved them by consensus.

The mechanism for the formation of the GDG varies by institution. In public institutions, clinical experts are generally appointed through institutional invitation addressed to the heads of the corresponding medical departments. On the other hand, methodologists are usually permanent staff of technology assessment offices or hired through external consultancy. In the private sector, a direct assignment is usually made by the medical management or the institution's quality committee. For statistical analysis, each participation in a CPG was considered as an independent record. If a professional participated in more than one guideline, each of their interventions was counted separately. This approach allows evaluating gender representation in all available positions in the GDG during the study period.

### Variables

For each CPG, the medical specialty (internal medicine, gynecology and obstetrics, surgery, or pediatrics), the publication date, and the institution responsible for its development were selected. For cases where a CPG addressed cross-cutting themes, a single primary specialty was assigned following two priority criteria: 1) the clinical department that requested or led the development, and 2) the main target population of the guideline. Two researchers (FCE and SCM) performed the classification independently; discrepancies were resolved by consensus or the intervention of a third researcher (CAD or WNG).

For each identified GDG member, gender and role (clinical physicians, methodologists, clinical reviewers, and methodological reviewers) were extracted. Gender identification was performed using the Genderize.io application (https://genderize.io), previously used in scientific articles ^13^^-^^15^. This tool assigns a probability score from 0 to 1. Additionally, the person explicitly identified in the CPG document as "coordinator," "leader," or "director" was defined as a leadership role. In cases where this position was shared by a man and a woman, it was categorized as mixed leadership. If the CPG did not explicitly state these positions, no leadership role was assigned for that group.

### Disparity

Gender disparity was evaluated following the recommendations of the National Healthcare Quality and Disparities Report (NHQDR) of 2023 ^16^^,^^17^. It recommends the use of two criteria to determine the differences between men and women: 1) the absolute difference between the priority population group and the reference group must be statistically significant with a p-value < 0.05 in a two-sided test; and 2) the relative difference between the priority population group and the reference group must be at least 10% (supplementary table 2).

### Statistical Methods

The data were analyzed using descriptive and analytical methods, reporting frequencies and percentages. Disparity was evaluated taking numerical parity (50%) as the benchmark for institutional equity. According to the NHQDR methodology, it is assumed that, in the absence of systemic barriers, the distribution of opportunities between genders should approximate equality of proportions. Therefore, the null hypothesis (H0) posited that there is no significant difference between the participation of men and women (p1=p2 =0.5), using this parameter as a technical measure to quantify the magnitude of the observed gender gap. A value of p<0.05 was considered statistically significant. The analysis was performed with R software (version 4.0.3), using the *prop.test()* function to compare observed and expected proportions.

### Ethical considerations

Being a study that employs the review of open-access CPG, ethics committee approval was not required.

## RESULTS

### Characteristics of the CPG included

**Figure 1:**
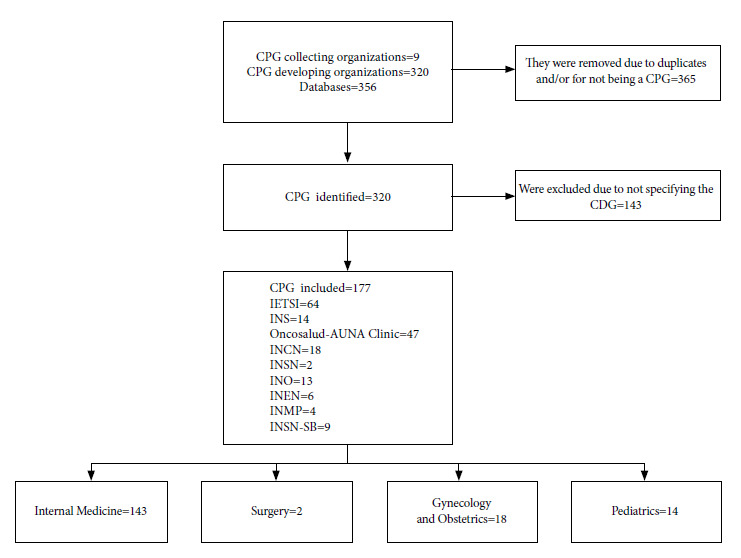
Flowchart of the selection process.

The search identified 320 CPG published between 2015 and July 30, 2024. 143 were excluded because they did not report information on their GDG members or did not allow the identification of roles and gender. Subsequently, 177 CPG that met the inclusion criteria were analyzed. The included CPG were issued by nine institutions. The Instituto de Evaluacion de Tecnologias en Salud e Investigacion (IETSI) published the largest number of CPG (36.2%; n=64), followed by Clinica Oncosalud-AUNA (26.5%; n=47) and the Instituto Nacional de Ciencias Neurologicas (INCN) (10.2%; n=18). The selection process is detailed in the flow diagram (figure 1). Most of the CPG were related to the specialty of internal medicine (80.8%; n=143). Among the 2597 GDG members, a gender disparity of 17.6% was observed (men [H]=58.8%; women [M]=41.2%; p<0.001) (table 1).

CPG: Clinical practice guidelines, CDG: CPG developing group, IETSI: Institute for Health Technology Assessment and Research, INS: National Institute of Health, INCN: National Institute of Neurological Sciences, INSN-Breña: National Institute of Child Health of Breña, INO: National Institute of Ophthalmology, INEN: National Institute of Neoplastic Diseases, INMP: National Maternal Perinatal Institute, INSN-SB: National Institute of the Child San Borja.

**Table 1:** Characteristics of Peruvian clinical practice guidelines by specialty, institution, role, methodology, and year.

Variables			Total n (%)	Men n (%)	Women n (%)	Difference in percentages (H - M)	p-value	Classification NHQDR
Gender			2597	1527 (58.8)	1070 (41,2)	17.6	<0.001	Disparity (W)
Role								
	Clinicians		1536	923 (60.1)	613 (39.9)	20,2	<0.001	Disparity (W)
	Methodologists		538	248 (46.1)	290 (53.9)	-7.8	<0,077	Advantage (B)
	Clinical reviewers		182	130 (71.4)	52 (28.6)	42.8	<0.001	Disparity (W)
	Methodologist Reviewers		60	50 (83.3)	10 (16.7)	66.6	<0.001	Disparity (W)
Medical specialty								
	Internal medicine		1697	1048 (61.8)	649 (38.2)	23.6	<0.001	Disparity (W)
		Cardiology	226	164 (72.6)	62 (27.4)	45.2	<0.001	Disparity (W)
		Endocrinology	41	28 (68.3)	13 (31.7)	36.6	<0.001	Disparity (W)
		Gastroenterology	82	61 (74.4)	21 (25,6)	48.8	<0.001	Disparity (W)
		Hematology	74	42 (56.8)	32 (43.2)	13.6	0.295	Equity (S)
		Infectious Diseases	36	25 (69.4)	11 (30.6)	38.8	0.030	Disparity (W)
		Internal Medicine	109	78 (71.6)	31 (28.4)	43.2	<0.001	Disparity (W)
		Nephrology	115	76 (66.1)	39 (33.9)	32.2	<0.001	Disparity (W)
		Pulmonology	58	36 (62.1)	22 (37.9)	24.2	0.088	Equity (S)
		Neurology	158	120 (75.9)	38 (24.1)	51.8	<0.001	Disparity
		Oncology	560	305 (54,5)	255 (45.5)	9	0.038	Disparity (W)
		Psychiatry	140	65 (46,4)	75 (53.6)	-7.2	0.447	Equity (S)
		Rheumatology	50	32 (64)	18 (36)	28	0.066	Equity (S)
		Others	48	16 (33.3)	32 (66.7)	-33,4	0.030	Advantage (B)
	Surgery		226	136 (60.2)	90 (39.8)	20.4	0.003	Disparity (W)
		Bariatric surgery	13	7 (53.8)	6 (46.2)	7.6	1	Equity (S)
		General Surgery	18	15 (83.3)	3 (16.7)	66.6	0.010	Disparity (W)
		Neurosurgery	4	2 (50)	2 (50)	0	1	Equity (S)
		Dentistry	32	19 (59.4)	13 (40.6)	18.8	0.377	Equity (S)
		Ophthalmology	133	71 (53.4)	62 (46.6)	6.8	0.488	Equity (S)
		Urology	26	22 (84.6)	4 (15.4)	69.2	<0.001	Disparity (W)
	Gynecology and Obstetrics		424	257 (60,6)	167 (39.4)	21,2	<0.001	Disparity (W)
	Pediatrics		241	86 (35.7)	155 (64.3)	-28.6	<0.001	Advantage (B)
		Neonatology	71	18 (25.4)	53 (74,6)	-49.2	<0.001	Advantage (B)
		Pediatrics	170	68 (40.0)	102 (60,0)	-20.0	0.011	Advantage (B)
Institution								
		IETSI	1004	645 (64.2)	359 (35.8)	28.4	<0.001	Disparity (W)
		INS	330	143 (43,3)	187 (56.7)	-13.4	0.018	Advantage (B)
		Clínica Oncosalud-AUNA	679	387 (57)	292 (43)	14	<0.001	Disparity (W)
		INCN	125	97 (77,6)	28 (22,4)	55.2	<0.001	Disparity (W)
		INSN - Breña	46	16 (34,8)	30 (65.2)	-30.4	0.055	Equity (S)
		INO	68	32 (47.1)	36 (52.9)	-5.8	0.716	Equity (S)
		INEN	130	70 (53.8)	60 (46,2)	7.6	0.430	Equity (S)
		INMP	180	126 (70)	54 (30)	40	<0.001	Disparity (W)
		INSN-SB	35	11 (31.4%)	24 (68.6%)	-37.2	0.043	Advantage (B)
Methodology for the Development of the CPG								
	GRADE		2316	1351 (58.3)	965 (41.7)	16.6	<0.001	Disparity (W)
	No GRADE		281	176 (62,6)	105 (37.4)	25.2	<0.001	Disparity (W)

H: men, M: women, CPG: Clinical practice guidelines, IETSI: Instituto de Evaluación de Tecnologías en Salud e Investigación, INS: Instituto Nacional de Salud, INCN: Instituto Nacional de Ciencias Neurológicas, INSN-Breña: Instituto Nacional de Salud del Niño de Breña, INO: Instituto Nacional de Oftalmología, INEN: Instituto Nacional de Enfermedades Neoplásicas, INMP: Instituto Nacional Materno Perinatal, INSN-SB: Instituto Nacional del Niño San Borja.. W: worse, S: same, B: better. NHQDR: National Healthcare Quality and Disparities Report, GRADE: Grading of Recommendations Assessment, Development and Evaluation.

### Gender differences by role in GDG

Of the 177 GDGs analyzed, 78 explicitly reported the presence of a leader or coordinator. In the evaluation of the leadership variable, a higher frequency of women was observed, who occupied this role in 60.2% (n=47) of cases; men represented 26.9% (n=21), and mixed leadership was identified in 12.8% (n=10). According to the NHQDR criteria, this role was classified into the «better» (*better*) category for female participation, showing a relative difference >10% and a p<0.05. Regarding the distribution of technical roles, women predominantly performed as methodologists (53.9%; n=290); however, given a relative difference less than 10% regarding parity, this role was operationalized under the «equity» (s*ame*) category. In contrast, technical gender «disparity» (*worse*) was confirmed in the remaining roles. In the clinical expert role, the relative disparity difference was 20.2% (H=60.1%, M=39.9%; p<0.001). This gap was more pronounced in review roles, with a relative disparity of 42.8% for clinical reviewers (H=71.4%, M=28.6%; p<0.001) and 66.6% for methodological reviewers (H=83.3%, M=16.7%; p<0.001) (table 1).

### Gender differences by specialty of the CPG

In the analysis by thematic areas, pediatrics stood out as the specialty with the highest female representation (64.3%); the subspecialty of neonatology reached a participation of 74.6%. Under the NHQDR criteria, pediatrics was classified in the category of «better» (*better*) for the female gender, by exceeding the 10% relative difference threshold in its favor (p<0.001).

In contrast, the four main specialties showed a technical gender «disparity» (*worse*). Internal medicine presented a relative difference of 38.2% (H=61.8%, M=38.2%; p<0.001). Clinical specialties with the largest relative gap included neurology (68.2%; H=75.9%, M=24.1%; p<0.001), gastroenterology (65.6%; H=74.4%, M=25.6%; p<0.001), cardiology (62.2%; H=72.6%, M=27.4%; p<0.001) and endocrinology (53.6%; H=68.3%, M=31.7%; p<0.001). Finally, surgery and gynecology and obstetrics also showed technical disparity, with relative differences of 33.9% (H=60.2%, M=39.8%; p<0.05) and 34.9% (H= 60.6%, M=39.4%; p<0.05) respectively (table 1).

### Gender differences by institution and year of publication

Regarding the institutions, technical gender disparity (w*orse*) was confirmed in IETSI and INCN with a relative difference of 44.2% (H=64.2%, M=35.8%; p<0.05) and 71.1% (H=77.6%, M=22.4%; p<0.05); correspondingly. In contrast, institutions such as INS, the Instituto Nacional de Salud del Niño (INSN) (Breña and San Borja), and the Instituto Nacional de Oftalmología (INO) were in the "equity" category (s*ame*), by showing relative differences below the 10% threshold or lacking statistical significance (p>0.05) (table 1).

**Figure 2:**
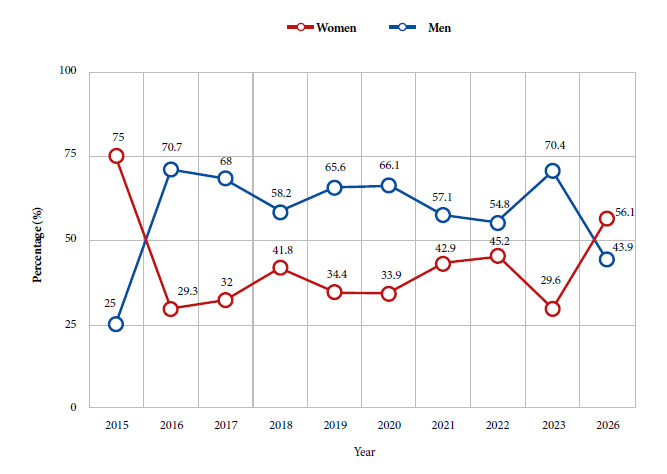
Percentage trends by gender in the development of clinical practice guidelines.

Regarding publication years, gender disparity was found in 2020 with a percentage difference of 32.2% (H=66.1%, M=33.9%; p<0.05). Similarly, gender disparity was also observed in the years 2016, 2017, 2018, 2019, 2021, and 2023 (figure 2).

### Gender difference according to the methodology for the development of CPG

In the CPG where the GRADE methodology was applied, a technical gender disparity (w*orse*) was identified, with a percentage difference of 16.6% (M=58.3%, F=41.7%; p<0.001) (table 1). This finding shows that, even the CPG developed with GRADE methodology, which standardize and formalize the panel formation process, show male participation above the established equity threshold.

## DISCUSSION

This study seeks to explore the presence of gender disparity in the development of CPG in Peru. According to our results, we identified female underrepresentation similar to that reported in other regions such as Australia, the United States, and the United Kingdom ^4^^,^^18^. This was consistent across the identified guidelines in relation to the roles performed, the topics, and the institutions where they were developed. Although in recent years the trend shows an increase in female participation, this growth varies throughout the analyzed period.

The findings show a female underrepresentation in roles such as clinicians and clinical reviewers. In the Peruvian context, the GDG formation process differs between these roles, suggesting that the causes of the disparity are multifactorial. It is relevant to highlight that, although operative leadership (coordination or direction of the GDG) falls predominantly on women (60.2%), this does not translate into equitable participation in the rest of the group's roles. This discrepancy suggests a «horizontal segregation» of functions. While women more frequently assume administrative and methodological leadership roles, men predominate as clinical experts and reviewers. Given that the incorporation of clinical experts is carried out through invitations to department heads, the disparity could be explained by the scarce presence of women occupying related roles. However, in the Peruvian context, female methodological leadership does not seem sufficient to break the gender gap in clinical board memberships, which depend on institutional structures external to the GDG itself.

On the other hand, it has been reported that gender influences the assignment of roles; generally, in scenarios where women lead committees, there tends to be greater female participation ^19^; however, in Peruvian CPG, this influence appears limited. In the case of clinical reviewers, whose selection is usually through open call, female underrepresentation could be associated with previous gaps in research trajectory ^20^ or a recruitment process that, despite being open, is still influenced by the gender of the evaluators ^19^.

Regarding medical specialty, pediatrics was the only field without significant disparity. This result aligns with global feminization trends in certain areas; for example, in the United States, the pediatrics specialty records 65% female participation. However, even in some pediatric subspecialties, such as pediatric surgery, gender disparity remains in favor of men ^21^^,^^22^.

Based on our findings, Peruvian public institutions that develop CPG registered a more pronounced gender disparity with male predominance compared to private organizations. This could be explained by hiring processes in the public sector, which usually follow the trend of male predominance ^23^. In Chile's public sector, as in Peru, the group that organizes the CPG selects GDG members based on pre-established roles ^24^. In contrast, in Spain, an open call is made to health professionals and scientific societies to list candidates who present the most suitable profiles. It should be noted that this methodology states that ideally the GEG should present a balance between sexes ^25^. The International Labour Organization extensively analyzes this issue and proposes as gender mainstreaming mechanisms the establishment of a diagnosis of the labor situation and intervention in the equal selection and hiring process ^26^. This includes changes such as characterizing each profile based on skills and work experience, omitting aspects linked to each gender; and promoting an equitable number of women and men applicants for the same position ^26^.

It is observed that at the beginning of the 2015-2024 period, women outnumbered men in their participation as part of the GDG; however, this differs over time. This phenomenon coincides with the «scissor effect», which explains that as a higher educational level is required or positions represent a higher hierarchy; women less frequently occupy these positions ^27^^,^^28^. This could coincide with the gradual implementation of the methodological guideline in Peru by the different institutions. This requires forming a GDG and including members who participate in decision-making (leadership), as well as being experts in the topics addressed (educational level).

The findings of this study can guide changes at different levels. First, institutions can use these results to promote gender equality in roles involved in CPG development. Second, greater reflection among researchers on this topic can contribute to fostering a more inclusive work environment and promoting the active participation of women in diverse teams. Third, GDG leaders, in both public and private organizations, can strengthen recruitment strategies to ensure balanced gender representation, including setting a minimum threshold for female participation in key roles. Finally, promoting equal participation in health decision-making processes, such as CPG development, can generate diverse and more inclusive recommendations that more accurately reflect the needs of the populations these guidelines are aimed at.

This study presents several limitations. First, the descriptive design does not allow establishing causal relationships, which limits the ability to identify the underlying factors contributing to the observed gender disparities. Second, the exclusion of certain clinical practice guidelines that did not detail the development groups could have slightly affected the results. Third, gender identification was performed using the Genderize.io tool. We acknowledge the limitations of this platform, which includes variable accuracy depending on the ethnic origin of names and a strictly binary classification that does not consider non-normative gender identities. To mitigate these biases, only results with a probability ≥0.9 were accepted.

In conclusion, Peruvian CPG published in the 2015-2024 period reveal significant gender disparity in GDG. While the female sex predominates in methodological roles and medical specialties such as pediatrics and neonatology, their participation is considerably lower in other roles and fields. Institutions such as the Instituto Nacional de Salud del Niño-San Borja (INSN-SB) show less disparity, while the INCN presents significant gaps. Although female representation increased in 2024, progress is not uniform across all areas. Given these findings, we recommend exploring this research objective using a qualitative design to understand the viewpoint and interaction among GDG members. Furthermore, the available literature describes heterogeneous methodologies for defining gender disparity. This article used a widely employed method, which can be considered as a suggestion for future research with similar approaches.
