# Supplementary material for: Gender disparities in the development of clinical practice guidelines in Peru
Source: Rev Peru Med Exp Salud Publica. 2026 Jun 25;43(2):171–8. doi: 10.17843/rpmesp.2026.432.15175 (PMC13456925; doi:10.17843/rpmesp.2026.432.15175)
Supplement: Supplementary file 2 [file rpmesp-43-02-15175-s001.docx]

**MATERIAL SUPLEMENTARIO**

**Lista de Abreviaciones:**

| **Sigla** | **Significado** |
| --- | --- |
| GPC | Guía(s) de práctica clínica |
| GEG | Grupo elaborador de la GPC |
| STROBE | *STrengthening the Reporting of OBservational studies in Epidemiology* |
| GRADE | *Grading of Recommendations Assessment, Development and Evaluation* |
| NHQDR | *National Healthcare Quality and Disparities Report* (Informe Nacional sobre Calidad y Disparidades en la Atención Médica) |
| STEM | Ciencia, tecnología, ingeniería y matemáticas (*Science, Technology, Engineering and Mathematics*) |
| UNESCO | *United Nations Educational, Scientific and Cultural Organization* |
| ICMJE | *International Committee of Medical Journal Editors* |
| IETSI | Instituto de Evaluación de Tecnologías en Salud e Investigación |
| INS | Instituto Nacional de Salud |
| INCN | Instituto Nacional de Ciencias Neurológicas |
| INSN-Breña | Instituto Nacional de Salud del Niño – Breña |
| INSN-SB | Instituto Nacional de Salud del Niño – San Borja |
| INO | Instituto Nacional de Oftalmología |
| INEN | Instituto Nacional de Enfermedades Neoplásicas |
| INMP | Instituto Nacional Materno Perinatal |
| Clínica Oncosalud-AUNA | Clínica oncológica privada de la red AUNA |

# **Tabla suplementaria 1**. Lista de búsqueda en organismos recopiladores, elaboradores, y base de datos de GPCs.

| **Organismos recopiladores de GPC:** | | | |
| --- | --- | --- | --- |
| Base Internacional de guias GRADE | <http://sites.bvsalud.org/bigg/biblio/> | Peru guidelines | 9 |
| **Organismos elaboradores** | | | |
| IETSI | <https://ietsi.essalud.gob.pe/gpc-guias-de-practica-clinica/> | Guías de práctica clínica | 64 |
| AUNA | <https://oncosalud.pe/documentos-interes/guias-de-practica-clinica> | Guías de práctica clínica | 47 |
| INEN | <https://portal.inen.sld.pe/guias-tecnicas/> | Guías de práctica clínica | 11 |
| INSN-SB | [https://portal.insnsb.gob.pe/guias-de-practica-clinicas/#](https://portal.insnsb.gob.pe/guias-de-practica-clinicas/) | Guías de práctica clínica | 139 |
| INSN | <http://www.insn.gob.pe/transparencia/normas-legales/guias-clinica?page=0> | Guías de práctica clínica | 2 |
| INS | <https://www.gob.pe/institucion/ins/informes-publicaciones/tipos/217-guias-de-practica-clinica> | Guías de práctica clínica | 14 |
| INO | <https://www.ino.gob.pe/documentos-tecnicos/#1628823248075-4ebf171f-b5c9> | Guías de práctica clínica | 13 |
| INMP | <https://www.inmp.gob.pe/institucional/guias-de-practica-clinica-y-de-procedimiento-del-departamento-en-neonatologia/1680205704> | Guías de práctica clínica | 4 |
| INCN | <https://www.incn.gob.pe/guias-de-practica-clinica-y-consentimientos-informados/> | Guías de práctica clínica | 26 |
| **Bases de datos:** | | | |
| PubMed | <https://www.ncbi.nlm.nih.gov/pubmed> | (Practice Guidelines as Topic OR Practice Guideline[Publication Type] OR ‘Practice Guideline’[tiab] OR ’Clinical practice guideline’[tiab] OR (Clinical*[tiab] AND Guideline*[tiab]) OR "Clinical guidelines"[Title/Abstract:~3] OR CGP[tiab] OR CGPs[tiab] OR Guideline*[tiab]) AND (Peru[Tiab] OR Peruvian[Tiab]) | 356 |
| Google Scholar | <https://scholar.google.com.pe/> | Peru AND guidelines | 100 |
| Google | <https://www.google.com> | - Peru AND guidelines - Guia de practica clinica AND Peru | 100 |
| TOTAL | | | 885 |

**Tabla Suplementaria 2. Matriz de Operacionalización de la Disparidad (Criterios AHRQ/NHQDR)**

| **Término** | **Definición y criterios técnicos** | **Clasificación final (NHQDR)** |
| --- | --- | --- |
| **Metodología de Evaluación** | Se aplican dos criterios simultáneos para determinar si la diferencia de participación entre géneros es sustantiva:  1. Significancia Absoluta: La diferencia entre el grupo prioritario (mujeres) y el de referencia (hombres) debe tener un valor p<0,05.  2. Significancia Relativa: debe ser de al menos el 10%. Se calcula mediante la fórmula ($[p1-p2]/p2\geq0.1$) donde p1 es la proporción alineada (aligned rate) del grupo prioritario (mujeres) y p2 es la proporción alineada del grupo de referencia (hombres).  El resultado debe ser ≥0,1 (equivalente al 10%) para confirmar que la diferencia tiene relevancia institucional y no es solo una fluctuación estadística menor. |  |
| **Disparidad** | El estimado del grupo prioritario (mujeres) es **menos favorable** que el del grupo de referencia (hombres) por al menos un 10% y con una p<0,05. | Peor (*worse*) |
| **Equidad / Paridad** | No existe diferencia estadísticamente significativa (p≥0,05) **o** la diferencia relativa es menor al 10%, considerándose una fluctuación estadística menor. | Igual (*same*) |
| **Ventaja de Participación** | El estimado del grupo prioritario (mujeres) es **más favorable** que el del grupo de referencia (hombres) por al menos un 10% y con una p<0,05. | Mejor (*better*) |
