# Supplementary material for: Gender disparities in the development of clinical practice guidelines in Peru
Source: Rev Peru Med Exp Salud Publica. 2026 Jun 25;43(2):171–8. doi: 10.17843/rpmesp.2026.432.15175 (PMC13456925; doi:10.17843/rpmesp.2026.432.15175)
Supplement: Translation

# Translation: Disparidades de género en la elaboración de guías de práctica clínica en Perú

**Authors:** Fabian A. Chavez-Ecos, Patricia J. Vera-Maccha, Sandra S. Chavez-Malpartida, Leonardo J. Uribe-Cavero, Alexander M. Parra-Huaroto, Ana M. Avalos-Bautista, Miguel A. Chavez-Gutarra, Carlos Alva-Díaz, Wendy Nieto-Gutierrez

**Affiliations:** 1 Grupo de investigación NEMECS: Neurociencias, Metabolismo, Efectividad Clínica y Sanitaria, Universidad Científica del Sur, Lima, Perú.; 2 Red de Cardiología y Salud Pública (RCSP), Ica, Perú.; 3 Sociedad Científica de Estudiantes de Medicina de Ica (SOCEMI), Facultad de Medicina Humana, Universidad Nacional San Luis Gonzaga, Ica, Perú.; 4 Facultad de Medicina Humana, Universidad Nacional Mayor de San Marcos, Lima, Perú.; 5 Servicio de Neurología, Departamento de Medicina y Oficina de Apoyo a la Docencia e Investigación (OADI), Hospital Daniel Alcides Carrión, Callao, Perú.; 6 Unidad de Investigación para la Generación de Síntesis de Evidencia en Salud, Vicerrectorado de Investigación, Universidad San Ignacio de Loyola, Lima, Perú.

**Keywords:** Equidad de Género, Guía de Práctica Clínica, Mujeres

## Abstract

**Objetivo.:**

Explorar la disparidad de género en la elaboración de guías de práctica clínica (GPC) en Perú.

**Materiales y métodos.:**

Se realizó un estudio descriptivo y observacional. Se realizó una búsqueda electrónica de las GPC peruanas publicadas desde el 2015 hasta el 30 de julio del 2024. Se empleó Microsoft Excel para la recolección de datos y RStudio para el análisis estadístico.

**Resultados.:**

Se analizaron 177 GPC publicadas entre el 2015 y el 2024 que reportaban información relacionada al grupo elaborador de la GPC (GEG). En general, los GEG incluyeron a 1527 hombres (58,8%) y 1070 mujeres (41,2%) entre expertos clínicos y metodólogos. Los hombres predominaron como expertos clínicos, sobre todo en especialidades como medicina interna y cirugía; mientras que las mujeres predominaron como metodólogas. La especialidad con mayor predominio masculino fue medicina interna (61,8%).

**Conclusiones.:**

El estudio reveló una menor participación femenina en el desarrollo de las GPC en Perú, así como una mayor disparidad de género en la participación en el ámbito quirúrgico y en instituciones públicas. La poca participación de mujeres en puestos de liderazgo influye en esta brecha. Se requieren estrategias para mejorar la equidad de género.

## INTRODUCCIÓN

A nivel mundial, la disparidad de género en la ciencia continúa latente ^1^. La UNESCO (United Nations Educational, Scientific and Cultural Organization) identificó en el año 2021 que el género femenino representa el 31,5% de los investigadores relacionados a la ciencia, tecnología, ingeniería y matemáticas (STEM) a nivel mundial, y 44,4% en Latinoamérica y el Caribe ^1^. Este desequilibrio está impulsado por factores socioculturales, como estereotipos de género, roles tradicionales y una percepción limitada de pertenencia en los campos STEM; fenómenos que inician desde temprana edad y pueden afectar la autosuficiencia y confianza de las mujeres que participan en estas áreas ^2^. Los datos disponibles sugieren una brecha de género acentuada en países en desarrollo, como Perú. El Censo Nacional de Investigación y Desarrollo de Centros de Investigación elaborado en 2016 mostró que el sexo femenino representaba la tercera parte de investigadores registrados ^3^. Por otra parte, en el ranking de los 200 investigadores peruanos con mayores indicadores bibliométricos se identificó que el 15% eran mujeres y solo una se encontraba en los primeros diez puestos ^4^. 

Esta problemática podría impactar en contextos clave como los procesos ligados a toma de decisiones en salud; dado que requieren de la participación de un grupo que sea representativo e inclusivo ^5^. El impacto de la disparidad de género en procesos como la elaboración de guías de práctica clínica (GPC) se ha descrito en países de altos ingresos. Por ejemplo, Australia registró una subrepresentación de mujeres en los paneles de desarrollo de GPC; el 41% de ellas participó como miembros y el 42% como desarrolladoras principales ^6^. En el Perú, las pautas metodológicas para la elaboración de GPC se publicaron el año 2015 y su implementación se ha consolidado de forma progresiva en los años posteriores. ^7^. Los datos de contribución según género en estos procesos sugieren una tendencia a la disparidad. Un estudio del año 2023 reveló que aproximadamente el 28% de los miembros participantes en la elaboración de GPC eran mujeres; aunque esta publicación se centró en guías de enfermedades cardiovasculares ^8^.

La disparidad de género tiene implicancias significativas para el grupo minoritario, como la reducción de oportunidades de educación, empleo y remuneración ^9^. Entender la magnitud de esta problemática en los procesos de toma de decisiones en salud es importante para identificar y abordar las necesidades del grupo minoritario desde un enfoque sensible al género ^10^. Esta perspectiva cobra especial relevancia en un contexto como el peruano, donde la violencia de género y la baja participación femenina en roles de liderazgo siguen siendo problemas latentes ^11^. El objetivo de este estudio es analizar la disparidad de género en la participación de la elaboración de GPC en el Perú.

**MENSAJES CLAVE**

Motivación para realizar el estudio. La disparidad de género constituye un tópico poco explorado en la investigación y los procesos de toma de decisiones en salud. Promover la igualdad en el desarrollo de guías de práctica clínica (GPC) puede orientar la generación de recomendaciones que reflejen una perspectiva inclusiva y diversa.

Principales hallazgos. Se identificó disparidad de género en los grupos elaboradores de GPC peruanas en relación de menor participación de las mujeres en los roles como expertas clínicas y revisoras.

Implicancias en salud pública. Concientizar sobre la disparidad de género en el desarrollo de las GPC puede generar la adopción de un enfoque institucional con alcance tanto público como privado.

## MATERIALES Y MÉTODOS

### Diseño de estudio y población

Se desarrolló un estudio del tipo descriptivo, y observacional de las GPC de Perú en base a la guía STROBE (STrengthening the Reporting of OBservational studies in Epidemiology) ^12^ para estudios observacionales para mejorar el reporte y la calidad del estudio.

### Procedimiento, criterios de inclusión, y exclusión

La selección de las GPC siguió un proceso estructurado. Primero, se consideraron todas las GPC publicadas a partir de 2015 (ya que este año fue el momento en que las GPC comenzaron a utilizar la metodología GRADE (Grading of Recommendations Assessment, Development and Evaluation) ^(^^7^. Segundo, se realizó una búsqueda electrónica sistemática en Google Scholar y plataformas relacionadas con instituciones y repositorios, tanto en español como en inglés (tabla suplementaria 1). Tercero, se incluyeron GPC de todos los temas médicos relacionados con medicina interna, obstetricia y ginecología, cirugía y pediatría.

Como criterio de exclusión, se descartaron aquellas GPC que no proporcionaban el listado nominal de los miembros del grupo elaborador de la guía (GEG) o cuya información fuera insuficiente para determinar el género y el rol de los participantes. Adicionalmente, si una GPC tenía múltiples versiones, se seleccionó la versión más reciente según la fecha de publicación. La información sobre el GEG se extrajo en una hoja de cálculo de Microsoft Excel 2019. Este proceso fue realizado por cuatro investigadores (PJVM, LJUC, AMPH y AMAB), y en caso de discrepancias, un quinto autor (CAD o WNG) las resolvió por consenso.

El mecanismo de conformación de los GEG varía según la institución. En las instituciones públicas, los expertos clínicos son generalmente designados mediante invitación institucional dirigida a las jefaturas de los departamentos médicos correspondientes. Por otro lado, los metodólogos suelen ser personal de planta de las oficinas de evaluación de tecnologías o contratados mediante consultoría externa. En el sector privado, habitualmente se da una asignación directa por parte de la dirección médica o el comité de calidad de la institución. Para el análisis estadístico, se consideró cada participación en una GPC como un registro independiente. Si un profesional participó en más de una guía, cada una de sus intervenciones fue contabilizada por separado. Este enfoque permite evaluar la representación de género en la totalidad de los puestos disponibles en los GEG durante el periodo de estudio.

### Variables

Para cada GPC, se seleccionó la especialidad médica (medicina interna, ginecología y obstetricia, cirugía o pediatría), la fecha de publicación y la institución responsable de su desarrollo. Para los casos donde una GPC abordaba temáticas transversales, se asignó una única especialidad primaria siguiendo dos criterios de prioridad: 1) el departamento clínico que solicitó o lideró la elaboración, y 2) la población objetivo principal de la guía. Dos investigadores (FCE y SCM) realizaron la clasificación de forma independiente; las discrepancias se resolvieron mediante consenso o la intervención de un tercer investigador (CAD o WNG).

Para cada miembro del GEG identificado, se extrajo el género y el rol (médicos clínicos, metodólogos, revisores clínicos y revisores metodológicos). La identificación de género se realizó mediante la aplicación Genderize.io (https://genderize.io), utilizada previamente en artículos científicos ^13^^-^^15^. Esta herramienta asigna una puntuación de probabilidad de 0 a 1. Adicionalmente, se definió como rol de liderazgo a la persona identificada explícitamente en el documento de la GPC como «coordinador», «líder» o «director». En los casos donde este cargo era compartido por un hombre y una mujer, se categorizó como liderazgo mixto. Si la GPC no explicitaba estos cargos, no se asignó rol de liderazgo para dicho grupo. 

### Disparidad

La disparidad de género se evaluó siguiendo las recomendaciones del National Healthcare Quality and Disparities Report (NHQDR) de 2023 ^16^^,^^17^. Este recomienda el uso de dos criterios para determinar las diferencias entre hombres y mujeres: 1) la diferencia absoluta entre el grupo de población prioritario y el grupo de referencia debe ser estadísticamente significativa con un valor de p<0,05 en una prueba bilateral; y 2) la diferencia relativa entre el grupo de población prioritario y el grupo de referencia debe ser al menos del 10% (tabla suplementaria 2).

### Métodos estadísticos

Los datos se analizaron mediante métodos descriptivos y analíticos, reportando frecuencias y porcentajes. La disparidad se evaluó tomando la paridad numérica (50%) como el punto de referencia de equidad institucional. De acuerdo con la metodología del NHQDR, se asume que, en ausencia de barreras sistémicas, la distribución de oportunidades entre géneros debería aproximarse a la igualdad de proporciones. Por tanto, la hipótesis nula (H0) postuló que no existe diferencia significativa entre la participación de hombres y mujeres (p1=p2 =0,5), utilizando este parámetro como una medida técnica para cuantificar la magnitud de la brecha de género observada. Un valor de p<0,05 se consideró como estadísticamente significativo. El análisis se realizó con el software R (versión 4.0.3), empleando la función *prop.test ()* para comparar las proporciones observadas y esperadas.

### Consideraciones éticas

Al ser un estudio que emplea la revisión de GPC de acceso abierto, no se requirió la aprobación por un comité de ética.

## RESULTADOS

### Características de las GPC incluidas

La búsqueda identificó 320 GPC publicadas entre 2015 y el 30 de julio de 2024. Se excluyeron 143 debido a que no reportaban información sobre los miembros de sus GEG o no permitían la identificación de los roles y género. Posteriormente, se analizaron 177 GPC que cumplían los criterios de inclusión. Las GPC incluidas fueron emitidas por nueve instituciones. El Instituto de Evaluación de Tecnologías en Salud e Investigación (IETSI) publicó el mayor número de GPC (36,2%; n=64), seguido de la Clínica Oncosalud-AUNA (26,5%; n=47) y el Instituto Nacional de Ciencias Neurológicas (INCN) (10,2%; n=18). El proceso de selección se detalla en el diagrama de flujo (figura 1). La mayoría de las GPC estaban relacionadas con la especialidad de medicina interna (80,8%; n=143). Entre los 2597 miembros de los GEG, se observó una disparidad de género del 17,6% (hombre [H]=58,8%; mujer [M]=41,2%; p<0,001) (tabla 1).

**Figura 1:** Flujograma del proceso de selección.

GPC: Guias de práctica clínica, GEG: grupo elaborador de la GPC, IETSI: Instituto de Evaluación de Tecnologías en Salud e Investigación, INS: Instituto Nacional de Salud, INCN: Instituto Nacional de Ciencias Neurológicas, INSN-Breña: Instituto Nacional de Salud del Niño de Breña, INO: Instituto Nacional de Oftalmología, INEN: Instituto Nacional de Enfermedades Neoplásicas, INMP: Instituto Nacional Materno Perinatal, INSN-SB: Instituto Nacional del Niño San Borja.

**Tabla 1:** Características de las guías de práctica clínica peruanas según especialidad, institución, rol, metodología y año.

Variables			Total n (%)	Hombres n (%)	Mujeres n (%)	Diferencia de porcentajes (H - M)	Valor de p	Clasificación NHQDR
Género			2597	1527 (58,8)	1070 (41,2)	17,6	<0,001	Disparidad (W)
Rol								
	Clínicos		1536	923 (60,1)	613 (39,9)	20,2	<0,001	Disparidad (W)
	Metodólogos		538	248 (46,1)	290 (53,9)	-7,8	<0,077	Ventaja (B)
	Revisores clínicos		182	130 (71,4)	52 (28,6)	42,8	<0,001	Disparidad (W)
	Revisores metodólogos		60	50 (83,3)	10 (16,7)	66,6	<0,001	Disparidad (W)
Especialidad médica								
	Medicina interna		1697	1048 (61,8)	649 (38,2)	23,6	<0,001	Disparidad (W)
		Cardiología	226	164 (72,6)	62 (27,4)	45,2	<0,001	Disparidad (W)
		Endocrinología	41	28 (68,3)	13 (31,7)	36,6	<0,001	Disparidad (W)
		Gastroenterología	82	61 (74,4)	21 (25,6)	48,8	<0,001	Disparidad (W)
		Hematología	74	42 (56,8)	32 (43,2)	13,6	0,295	Equidad (S)
		Infectología	36	25 (69,4)	11 (30,6)	38,8	0,030	Disparidad (W)
		Medicina Interna	109	78 (71,6)	31 (28,4)	43,2	<0,001	Disparidad (W)
		Nefrología	115	76 (66,1)	39 (33,9)	32,2	<0,001	Disparidad (W)
		Neumología	58	36 (62,1)	22 (37,9)	24,2	0,088	Equidad (S)
		Neurología	158	120 (75,9)	38 (24,1)	51,8	<0,001	Disparidad (W)
		Oncología	560	305 (54,5)	255 (45,5)	9	0,038	Disparidad (W)
		Psiquiatría	140	65 (46,4)	75 (53,6)	-7,2	0,447	Equidad (S)
		Reumatología	50	32 (64)	18 (36)	28	0,066	Equidad (S)
		Otros	48	16 (33,3)	32 (66,7)	-33,4	0,030	Ventaja (B)
	Cirugía		226	136 (60,2)	90 (39,8)	20,4	0,003	Disparidad (W)
		Cirugía bariátrica	13	7 (53,8)	6 (46,2)	7,6	1	Equidad (S)
		Cirugía general	18	15 (83,3)	3 (16,7)	66,6	0,010	Disparidad (W)
		Neurocirugía	4	2 (50)	2 (50)	0	1	Equidad (S)
		Odontología	32	19 (59,4)	13 (40,6)	18,8	0,377	Equidad (S)
		Oftalmología	133	71 (53,4)	62 (46,6)	6,8	0,488	Equidad (S)
		Urología	26	22 (84,6)	4 (15,4)	69,2	<0,001	Disparidad (W)
	Ginecología y Obstetricia		424	257 (60,6)	167 (39,4)	21,2	<0,001	Disparidad (W)
	Pediatría		241	86 (35,7)	155 (64,3)	-28,6	<0,001	Ventaja (B)
		Neonatología	71	18 (25,4)	53 (74,6)	-49,2	<0,001	Ventaja (B)
		Pediatría	170	68 (40,0)	102 (60,0)	-20,0	0,011	Ventaja (B)
Institución								
		IETSI	1004	645 (64,2)	359 (35,8)	28,4	<0,001	Disparidad (W)
		INS	330	143 (43,3)	187 (56,7)	-13,4	0,018	Ventaja (B)
		Clínica Oncosalud-AUNA	679	387 (57)	292 (43)	14	<0,001	Disparidad (W)
		INCN	125	97 (77,6)	28 (22,4)	55,2	<0,001	Disparidad (W)
		INSN - Breña	46	16 (34,8)	30 (65,2)	-30,4	0,055	Equidad (S)
		INO	68	32 (47,1)	36 (52,9)	-5,8	0,716	Equidad (S)
		INEN	130	70 (53,8)	60 (46,2)	7,6	0,430	Equidad (S)
		INMP	180	126 (70)	54 (30)	40	<0,001	Disparidad (W)
		INSN-SB	35	11 (31,4)	24 (68,6)	-37,2	0,043	Ventaja (B)
Metodología para la Elaboración de la GPC								
	GRADE		2316	1351 (58,3)	965 (41,7)	16,6	<0,001	Disparidad (W)
	No GRADE		281	176 (62,6)	105 (37,4)	25,2	<0,001	Disparidad (W)

H: hombres, M: mujeres, GPC: Guias de práctica clínica, IETSI: Instituto de Evaluación de Tecnologías en Salud e Investigación, INS: Instituto Nacional de Salud, INCN: Instituto Nacional de Ciencias Neurológicas, INSN-Breña: Instituto Nacional de Salud del Niño de Breña, INO: Instituto Nacional de Oftalmología, INEN: Instituto Nacional de Enfermedades Neoplásicas, INMP: Instituto Nacional Materno Perinatal, INSN-SB: Instituto Nacional del Niño San Borja. W: worse, S: same, B: better. NHQDR: National Healthcare Quality and Disparities Report, GRADE: Grading of Recommendations Assessment, Development and Evaluation.

### Diferencias de género según el rol en los GEG

De los 177 GEG analizados, 78 reportaron explícitamente la presencia de un líder o coordinador. En la evaluación de la variable liderazgo, se observó una mayor frecuencia de mujeres, quienes ocuparon este rol en el 60,2% (n=47) de los casos; los hombres representaron el 26,9% (n=21) y se identificó un liderazgo mixto en el 12,8% (n=10). De acuerdo con los criterios del NHQDR, este rol se clasificó en la categoría de «mejor» (*better*) para la participación femenina al presentar una diferencia relativa >10% y un p<0,05. Respecto a la distribución de roles técnicos, las mujeres se desempeñaron mayoritariamente como metodólogas (53,9%; n=290); sin embargo, al presentar una diferencia relativa menor al 10% respecto a la paridad, este rol se operacionalizó bajo la categoría de «equidad» (s*ame*). En contraste, se confirmó «disparidad» de género técnica (*worse*) en los roles restantes. En el rol de experto clínico, la diferencia relativa de disparidad fue del 20,2% (H=60,1%, M=39,9%; p<0,001). Esta brecha fue más pronunciada en los roles de revisión, con una disparidad relativa del 42,8% para revisores clínicos (H=71,4%, M=28,6%; p<0,001) y del 66,6% para revisores metodológicos (H=83,3%, M=16,7%; p<0,001) (tabla 1).

### Diferencias de género por especialidad de la GPC

En el análisis por áreas temáticas, pediatría destacó como la especialidad con mayor representación femenina (64,3%); la subespecialidad de neonatología alcanzó una participación del 74,6%. Bajo los criterios del NHQDR, pediatría se clasificó en la categoría de «mejor» (*better*) para el género femenino, al superar el umbral de diferencia relativa del 10% a su favor (p<0,001).

En contraste, las cuatro especialidades principales mostraron una «disparidad» de género técnica (*worse*). Medicina interna presentó una diferencia relativa del 38,2% (H=61,8%, M=38,2%; p<0,001). Las especialidades clínicas con mayor brecha relativa incluyeron neurología (68,2%; H=75,9%, M=24,1%; p<0,001), gastroenterología (65,6%; H=74,4%, M=25,6%; p<0,001), cardiología (62,2%; H=72,6%, M=27,4%; p<0,001) y endocrinología (53,6%; H=68,3%, M=31,7%; p<0,001). Finalmente, cirugía y ginecología y obstetricia también evidenciaron disparidad técnica, con diferencias relativas del 33,9% (H=60,2%, M=39,8%; p<0,05) y 34,9% (H= 60,6%, M=39,4%; p<0,05) respectivamente (tabla 1).

### Diferencias de género por institución y año de publicación

Respecto a las instituciones, se confirmó disparidad de género técnica (w*orse*) en el IETSI y en el INCN con una diferencia relativa del 44,2% (H=64,2%, M=35,8%; p<0,05) y 71,1% (H=77,6%, M=22,4%; p<0,05); correspondientemente. Por el contrario, instituciones como el INS, el Instituto Nacional de Salud del Niño (INSN) (Breña y San Borja) y el Instituto Nacional de Oftalmología (INO) se situaron en la categoría de «equidad» (s*ame*), al presentar diferencias relativas inferiores al umbral del 10% o carecer de significancia estadística (p>0,05) (tabla 1).

En cuanto a los años de publicación, en 2020 se encontró disparidad de género con una diferencia porcentual del 32,2 % (H=66,1 %, M=33,9 %; p<0,05). De igual manera, también se observó disparidad de género en los años 2016, 2017, 2018, 2019, 2021 y 2023 (figura 2).

**Figura 2:** Tendencias de porcentajes según género en la elaboración de guías de práctica clínica.

### Diferencia de género según la metodología para la elaboración de GPC

En las GPC donde se aplicó la metodología GRADE, se identificó una disparidad de género técnica (w*orse*), con una diferencia porcentual de 16,6% (H=58,3%, M=41,7%; p<0,001) (tabla 1). Este hallazgo muestra que, incluso las GPC elaboradas con metodología GRADE, que estandarizan y formalizan el proceso de conformación del panel, muestran una participación masculina por encima del umbral de equidad establecido.

## DISCUSIÓN

Este estudio busca explorar la presencia de disparidad de género en el desarrollo de GPC en Perú. Según nuestros resultados, identificamos una subrepresentación femenina similar a la reportada en otras regiones como Australia, Estados Unidos y el Reino Unido ^4^^,^^18^. Esta fue consistente en las guías identificadas en relación a los roles desempeñados, las temáticas y las instituciones donde se desarrollaron. Si bien en los últimos años la tendencia muestra un aumento de la participación femenina, este crecimiento varía a lo largo del período analizado.

Los hallazgos muestran una subrepresentación femenina en roles como clínicos y revisores clínicos. En el contexto peruano, el proceso de conformación del GEG difiere entre estos roles, lo que sugiere que las causas de la disparidad son multifactoriales. Es relevante destacar que, aunque el liderazgo operativo (coordinación o dirección del GEG) recae mayoritariamente en mujeres (60,2%), esto no se traduce en una participación equitativa en el resto de los roles del grupo. Esta discrepancia sugiere una «segregación horizontal» de funciones. Mientras que las mujeres asumen con mayor frecuencia roles de liderazgo administrativo y metodológico, los hombres predominan como expertos clínicos y revisores. Dado que la incorporación de expertos clínicos se realiza mediante invitaciones a los jefes de departamentos, la disparidad podría explicarse por la escasa presencia de mujeres que ocupen roles relacionados. Sin embargo, en el contexto peruano, el liderazgo metodológico femenino parece no ser suficiente para romper la brecha de género en las vocalías clínicas, las cuales dependen de estructuras institucionales externas al propio GEG.

Por otro lado, se ha reportado que el género influye en la designación de funciones; generalmente, en escenarios donde las mujeres lideran comités suele haber una mayor participación femenina ^19^; no obstante, en las GPC peruanas, esta influencia parece limitada. En el caso de los revisores clínicos, cuya selección suele ser por convocatoria abierta, la subrepresentación femenina podría estar asociada a brechas previas en la trayectoria de investigación ^20^ o a un proceso de reclutamiento que, a pesar de ser abierto, sigue influenciado por el género de los evaluadores ^19^.

En cuanto a la especialidad médica, pediatría fue el único campo sin disparidad significativa. Este resultado concuerda con las tendencias globales de feminización en ciertas áreas; por ejemplo, en Estados Unidos, la especialidad de pediatría registra un 65% de participación femenina. Sin embargo, incluso en algunas subespecialidades pediátricas, como cirugía pediátrica, la disparidad de genero se mantiene a favor de los hombres ^21^^,^^22^.

En base a nuestros hallazgos, las instituciones públicas peruanas que desarrollan GPC registraron una disparidad de género con predominio masculino más pronunciada en comparación de las organizaciones privadas. Esto podría explicarse por los procesos de contratación en el sector público, los cuales suelen seguir la tendencia de predominio masculino ^23^. En el sector público de Chile, al igual que en el Perú, el grupo que organiza la GPC selecciona a los miembros del GEG en base a los roles preestablecidos ^24^. En cambio, en España, se realiza una convocatoria abierta a profesionales del ámbito sanitario y sociedades científicas para enlistar candidatos que presenten los perfiles más adecuados. Cabe resaltar que esta metodología declara que idealmente el GEG debe presentar equilibrio entre sexos ^25^. La Organización Internacional de Trabajo analiza esta problemática ampliamente y plantea como mecanismos de enfoque de género el establecer un diagnóstico de la situación laboral e intermediar en el proceso de selección y contratación igualitaria ^26^. Esto incluye cambios como el caracterizar cada perfil en base a capacidades y experiencia laboral, omitiendo aspectos ligados a cada género; y que se promueva el contar con un numero equitativo de mujeres y hombres postulantes a un mismo cargo ^26^.

Se observa que al inicio del periodo 2015-2024 las mujeres superaban a los hombres en su participación como parte de los GEG; sin embargo, esto difiere en el transcurso del tiempo. Este fenómeno coincide con el «efecto tijera», el cual explica que a medida que se requiera un mayor nivel educativo o representen mayor jerarquía; las mujeres ocupan con menos frecuencia estos cargos ^27^^,^^28^. Esto podría coincidir con la implementación gradual que se ha dado de la pauta metodológica en el Perú por las distintas instituciones. Esta exige conformar un GEG e incluir a miembros que participen en la toma de decisiones (liderazgo), así como también, sean expertos en las temáticas abordadas (nivel educativo).

Los hallazgos de este estudio pueden orientar cambios a distintos niveles. En primer lugar, las instituciones pueden utilizar estos resultados para promover la igualdad de género en los roles involucrados en el desarrollo de GPC. En segundo lugar, una mayor reflexión entre los investigadores sobre esta temática puede contribuir a fomentar un entorno laboral más inclusivo y fomentar la participación activa de las mujeres en diversos equipos. En tercer lugar, los líderes de los GEG, tanto en organizaciones públicas como privadas, pueden fortalecer las estrategias de reclutamiento para garantizar una representación de género equilibrada, incluyendo el establecimiento de un umbral mínimo para la participación femenina en roles clave. Finalmente, promover la participación igualitaria en los procesos de toma de decisiones en materia de salud, como el desarrollo de GPC, puede generar recomendaciones diversas y más inclusivas que reflejen con mayor precisión las necesidades de las poblaciones a las que estas guías están dirigidas.

Este estudio presenta varias limitaciones. En primer lugar, el diseño descriptivo no permite establecer relaciones causales, lo que limita la capacidad de identificar los factores subyacentes que contribuyen a las disparidades de género observadas. En segundo lugar, la exclusión de ciertas guías de práctica clínica que no detallaban los grupos de desarrollo podría haber afectado ligeramente los resultados. En tercer lugar, la identificación de género se realizó mediante la herramienta Genderize.io. Reconocemos las limitaciones de esta plataforma, que incluye una precisión variable según el origen étnico de los nombres y una clasificación estrictamente binaria que no contempla identidades de género no normativas. Para mitigar estos sesgos, solo se aceptaron resultados con una probabilidad ≥0,9.

En conclusión, las GPC peruanas publicadas en el periodo 2015-2024 revelan una disparidad de género significativa en los GEG. Mientras que el sexo femenino predomina en roles metodológicos y especialidades médicas como pediatría y neonatología, su participación es considerablemente menor en otros roles y campos. Instituciones como el Instituto Nacional de Salud del Niño-San Borja (INSN-SB) muestran menor disparidad, mientras que el INCN presenta importantes brechas. Si bien, la representación femenina aumentó en 2024, el progreso no es uniforme en todas las áreas. Dados estos hallazgos, recomendamos explorar este objetivo de investigación utilizando un diseño cualitativo para comprender el punto de vista y la interacción entre los miembros de los GEG. Además, la literatura disponible describe metodologías heterogéneas para definir la disparidad de género. Este artículo utilizó un método ampliamente empleado, que puede considerarse como sugerencia para futuras investigaciones con enfoques similares.
